# Cleaning and disinfection of the root canal system provided by four active supplementary irrigation methods

**DOI:** 10.1038/s41598-024-53375-8

**Published:** 2024-02-15

**Authors:** Alessandra Timponi Goes Cruz, Adriane Antoniw Klemz, Edvaldo Antônio Ribeiro Rosa, Fabiana Soares Grecca, Bianca Mattos, Lucila Piasecki, Ricardo Machado, Sérgio Aparecido Ignácio, Ulisses Xavier da Silva Neto

**Affiliations:** 1 Department of Endodontics, College of Dentistry, Pontifical Catholic University of Paraná–PUC/PR, Curitiba, Paraná Brazil; 2 Department of Biosciences, College of Dentistry, Pontifical Catholic University of Paraná–PUC/PR, Curitiba, Paraná Brazil; 3 Department of Endodontics, College of Dentistry, Federal University of Rio Grande do Sul–UFRGS, Porto Alegre, Rio Grande do Sul Brazil; 4 Department of Periodontics & Endodontics, College of Dentistry, University at Buffalo, Buffalo, NY USA; 5 Clinical practice limited to Endodontics, Navegantes, Santa Catarina Brazil; 6 Department of Statistics, College of Dentistry, Pontifical Catholic University of Paraná–PUC/PR, Curitiba, Paraná Brazil

**Keywords:** Dentistry, Endodontics, Endodontic instruments, Root canal treatment

## Abstract

This in vitro study evaluated the bacterial reduction provided by the EndoActivator (EA), Easy Clean (EC), passive ultrasonic irrigation (PUI), and XP-Endo Finisher. Eight-four mesial roots of mandibular first molars were instrumented, inoculated with *Enterococcus faecalis*, and divided into four groups (n. 20). Bacterial reduction in the main canals and dentinal tubules were respectively determined by MTT assays and Live/Dead BackLight technique through confocal laser scanning microscopy (CLSM) at 50, 100, and 150 µm in-depth (n. 10 per group). Statistical analyses were conducted following a significance level of 95% (P < 0.05). A significant statistical difference was just identified between XPF and EC in the main canals. In the dentinal tubules from the main root canals, at 100 and 150 µm in-depths, significant statistical differences were only observed between XPF and EC (P = 0.027) for the former and between XPF and EC (P = 0.011) and XPF and PUI (P = 0.021) for the latter. In the dentinal tubules from the isthmus, at 100 µm in-depth, statistically relevant differences did occur between XPF and EC (P = 0.038) and EC and EA (P = 0.029). At 150 µm in-depth, these differences were only significant by comparing XPF and PUI (P = 0.025) and XPF and EC (P = 0.036). Although no irrigation method could thoroughly disinfect the RCS, bacterial reduction indexes were generally better after using XPF.

## Introduction

Chemomechanical preparation is the main responsible for the bacterial load reduction in the root canal system (RCS) to prevent the development or promote conditions for the healing of a perirradicular disease. It is primarily based on the associated use of endodontic instruments (mechanical cleaning) and auxiliary chemical solutions (chemical cleaning), further complemented by the physical cleaning performed by injection followed by aspiration of the irrigating solutions into the RCS^1,2^.

The metallurgical revolution occurred in the last years has positively impacted endodontic science, stemming from the development and improvement of instruments with different designs, objectives, kinematics, characteristics, and properties^3^. However, the difficulties imposed by the anatomical complexity and the infection of the RCS continue to be the pillars of endodontic failure^4^, thus encouraging the study of alternatives to enhance the chemical and disinfection processes^5–9^.

The EndoActivator system (EAS) (Dentsply-Maillefer, Ballaigues, Switzerland) is a sonically driven activation tool developed to promote a vigorous agitation of the irrigant or chelating solutions within the root canals. Using a needle and syringe, the system has raised irrigation efficiency better than conventional irrigation (CI). It comprises a portable handpiece and three disposable flexible polymer tips of distinct sizes (15/02, 25/04, and 35/04) that do not wear the root canal walls^10,11^.

Easy Clean (EC) is an acrylonitrile butadiene styrene plastic file (Easy Equipamentos Odontológicos, Belo Horizonte, Minas Gerais, Brazil). It has a size of 25/04 and an “aircraft wing” shaped cross-section that may be used in both reciprocating or rotary motion to enhance endodontic cleaning and disinfection^12,13^.

Passive ultrasonic irrigation (PUI) was initially described by Weller et al.^14^ and is based on the passive insertion of a metal tip/file attached to an ultrasonic device oscillating at a frequency of 30 kHz into a canal filled with irrigating or chelating solutions^14^. When the instrument is activated, it is surrounded by acoustic streaming to boost the performance of the agitation of the irrigating or chelating solutions, increasing the endodontic cleaning and disinfection^15^.

XP-Endo Finisher (XPF) (FKG Dentaire, La Chaux-de-Fonds, Switzerland) is an n. 25 non-tapered instrument produced with a special NiTi alloy called MaxWire (Martensite-Austenite Electropolish-Flex, FKG). The file is straight in its martensite phase (at room temperature and when cooled) and changes to its austenite phase when exposed to the body temperature. From this moment on, it takes on a unique spoon shape, 10 mm long from the tip and 1.5 mm deep, fashioned by its molecular memory. The instrument should be used at 800 rpm, after an apical enlargement of at least n. 25, and with the canal filled with irrigating or chelating solutions to increase the endodontic cleaning and disinfection processes^12,16^.

Only a few studies have been conducted to investigate the cleaning and disinfection of the RCS (including the dentinal tubules), comparing different active supplementary irrigation methods in teeth and/or roots with well-known anatomical complexity. Therefore, this research aimed to evaluate the performance of EA, EC, PUI, and XPF on the bacterial reduction in the main root canals and dentinal tubules from root canals and isthmus in the mesial roots of extracted human mandibular first molars^17,18^. The null hypothesis established was that there is no difference in the cleaning and disinfection potential among the methods investigated herein, regardless of the place (main root canals and dentinal tubules from the main root canals and isthmus).

## Methods

### Approval by the research ethics committee

For performing this scientific investigation, a research project was previously written, submitted, and duly approved by the Research Ethics Committee of the Pontifical Catholic University of Paraná–PUC/PR, Curitiba, Paraná, Brazil (CAAE. 61195016.5.0000.0020) on 03/05/2018. Nonetheless, all stages exposed below were strictly performed following the Declaration of Helsinki^19^.

### Sample selection (first stage)

Informed consent was obtained from all subjects and/or their legal guardian(s) to use their extracted teeth for scientific purposes. Then, the Human Teeth Bank of the University provided three hundred and sixty human mandibular first molars extracted for reasons unrelated to the present study. After, they were digitally radiographed in two directions (buccolingual and mesiodistal) and then subjected to cone-beam computed tomography (CBCT) scanning process for the initial assessment of their internal anatomy using a Scanora 3D tomograph (Soredex, Tuusula, Finland) set with the following parameters: 120 kV, 12.5 mA, a field of view (FOV) measuring 75 × 100 mm, and a voxel size of 0.2 mm. This initial analysis permitted the preliminary selection of 180 specimens based on the subsequent criteria: no prior endodontic treatment, absence of pulp calcifications, and complete root formation without significant root curvatures (falling within 20° to 75°)^20^. During this initial phase of sample selection, exclusion criteria consisted of endodontically treated teeth, teeth with pulp calcifications, open apexes, or teeth displaying pronounced root curvatures (greater than or equal to 75°)^20^.

### Sample selection (second stage)

The 180 specimens previously chosen underwent a micro-computed tomography (micro-CT) scanning process using the SkyScan 1174 system (Bruker micro-CT, Kontich, Belgium). This assessment aimed to confirm the absence of cracks, fractures, or root resorptions. Additionally, it provided more detailed information regarding the internal root canal anatomy, particularly in the mesial roots, which would have to present with two distinct canals that converged into a single foramen through a cervical isthmus, conforming to the Vertucci Type II classification (additional inclusion criterion). The technical parameters for the micro-CT scanning process were as follows: 800 μA, 50 kVp, rotation step of 0.7° spanning 360° around the vertical axis, pixel size of 16.82 μm, and 0.5-mm thick aluminum filter. The time for scanning each specimen was around 55 min. Then, three-dimensional images were generated, and volumetric analyses were carried out using the NRecon V.1.6.8.0 and CTAn v. 1.12 softwares (Bruker micro-CT)^10^. At − 1 mm from the apical foramen, the canals also would have the following values, represented as mean ± standard deviation (median)—area (mm^2^): 0.191 ± 0.13 (0.157); perimeter (mm): 2.687 ± 0.84 (2.245); roundness: 0.403 ± 0.20 (0.093); major diameter (mm): 0.857 ± 0.42 (0.291), and minor diameter: 0.325 ± 0.13 (0.269) (complementary inclusion criteria)^21^. During this second stage of sample selection, the additional exclusion criteria included teeth with a mesial configuration differing from the Vertucci Type II classification or teeth with mesial roots and canals at the 1 mm mark from the apical foramen displaying values of area, perimeter, roundness, major and minor diameters that differed from those previously specified^21^. As a result, 84 specimens presenting stringent anatomical features in the mesial root were definitively selected to constitute the sample of the current research.

### Specimens’ preparation

Initially, the teeth were submitted to hemi-sections using a low-speed steel cutting disc (Isomet-Buehler, Lake Bluff, Illinois, United States). After discarding the distal portion, each specimen's pulp cavity coronary distal wall was reconstructed with composite resin (Opallis, FGM, Joinville, Santa Catarina, Brazil) to create a reservoir for the auxiliary chemical solutions. Afterward, the crown was cut using a low-speed steel cutting disc (Isomet-Buehler) to obtain specimens with a standard length of 17 mm. The working length (WL) was established by inserting a n. 15K-file (Dentsply-Maillefer) until the apical foramen, subtracting 1 mm from this measurement to establish the apical limit around the apical constriction^22^. This procedure was performed with an operating microscope (DF Vasconcellos, Londrina, Paraná, Brazil) at 8× magnification.

Biomechanical preparation was performed using the Medium File (35/06) of the WaveOne Gold system (Dentsply-Maillefer) powered by an X-Smart Plus electric endodontic motor (Dentsply-Maillefer) according to the manufacturer's instructions. After each instrument use, the apical patency was checked with an n. 10K-file (Dentsply-Maillefer). Irrigation was carried out with 2.5 ml of 2.5% sodium hypochlorite (NaOCl) solution (Farmácia Precisão, Curitiba, Paraná, Brazil) using a 30-G NaviTip needle (Ultradent, South Jordan, UT, USA) calibrated at − 4 mm from the WL. After the chemomechanical preparation, the final irrigation was performed with 2.5 ml of 2.5% NaOCl (Farmácia Precisão), followed by the same amount of both 17% EDTA and 2.5% NaOCl (Farmácia Precisão). Thus, all root canals were irrigated with the same quantity of NaOCl (12.5 ml) and EDTA (2.5 ml). Complete drying of the canals was achieved using WaveOne Gold Medium absorbent paper points (Dentsply-Maillefer), and the roots were then covered by two layers of nail enamel, leaving only the foraminal region free to prevent the contamination of the canals. The teeth were autoclaved at 121 °C and immersed in a phosphate-buffered solution (PBS).

### Canal inoculation with* Enterococcus faecalis*

A standard suspension of *Enterococcus faecalis (E. faecalis)* (ATCC 29212™) was created in a concentration of 1 × 10^8^ cells/ml. This suspension was prepared from a 24-h bacterial culture in brain heart infusion broth (BHI). Using sterile 1 ml insulin syringes equipped with a 30-gauge needle, 40 specimens were filled up to the orifice level with the *E. faecalis* suspension. Subsequently, they were placed in 15 ml tubes containing 10 ml of BHI broth and incubated at 37 °C for 21 days in an environment with 100% humidity, favoring the bacterial growth and the colonization of the RCS (main root canal and dentinal tubules). Every three days, BHI broth was replaced^17^.

### Supplementary disinfection methods

After this period, specimens were removed from the inoculation tubes, and the root apexes were sealed with composite resin in a clean environment with a laminar flow cabinet to prevent contamination. Then, they were randomly divided into four groups (n. 10) according to the active supplementary disinfection methods investigated (Table 1).

**Table 1:** Active supplementary disinfection methods and respective descriptions.

Groups	Descriptions
EA	Filling the root canals with 2.5 ml of 2.5% NaOCl (Farmácia Precisão) using a NaviTip 31‑gauge double side-port needle (Ultradent) followed by the insertion of an EA medium tip (25/04) (Dentsply-Maillefer) at − 1 mm from the WL at 10000 cycles per minute powered by the system handpiece for 60 s. The same procedures were performed with 2.5 ml of 17% EDTA (Farmácia Precisão)
EC	Filling the root canals with 2.5 ml of 2.5% NaOCl (Farmácia Precisão) using a NaviTip 31‑gauge double side-port needle (Ultradent), followed by the introduction of an EC (Easy Equipamentos Odontológicos) at − 1 mm of the WL, activated by an electric endodontic motor XSmart Plus (Dentsply-Maillefer) in reciprocating motion (WaveOne ALL) for 60 s. The same procedures were performed with 2.5 ml of 17% EDTA (Farmácia Precisão)
PUI	Filling the root canals with 2.5 ml of 2.5% NaOCl using a NaviTip 31‑gauge double side-port needle (Ultradent), followed by the introduction of a 20/01 ultrasonic irrigation file (Irrisonic E1, Helse, Santa Rosa de Viterbo, São Paulo, Brazil), calibrated at − 1 mm of the WL, adjusted on 15% of power (Endodontics mode) powered by an ultrasonic device (Jet Sonic, Gnatus, São Paulo, São Paulo, Brazil) for 60 s. The same procedures were performed with 2.5 ml of 17% EDTA (Farmácia Precisão)
XPF	Filling the root canals with 2.5 ml of 2.5% NaOCl (Farmácia Precisão) using a NaviTip 31‑gauge double side-port needle (Ultradent), followed by the insertion of an XPF (FKG) at − 1 mm of the WL activated by an X-Smart Plus electric endodontic motor (Dentsply-Maillefer) in continuous rotary motion (800 rpm/1 N.cm) for 60 s. Before insertion into the root canal, the instrument was cooled with a cooling spray (Endo-Frost, Roeko, Langenau, Germany). The same procedures were performed with 2.5 ml of 17% EDTA (Farmácia Precisão)

The final toilette was performed passively for all specimens using 2.5 ml of 2.5% NaOCl and 2.5 ml of 10% sodium thiosulfate (Farmácia Precisão).

### *E. faecalis* sampling

Following the 21 days of bacterial inoculation, samples of *E. faecalis* from the root canals of each specimen were procured before (S1) and after (S2) the disinfection procedures. All materials and instruments employed in the subsequent sample retrieval were thoroughly sterilized.

PBS replaced the BHI broth in the root canals for the collection of S1 samples. Then, a n. 25 Hedstroem instrument (Dentsply-Maillefer) was used to file the dentinal walls with 20 vigorous strokes. The canal contents were aspirated using a 1 ml insulin syringe with a 25-gauge needle and transferred into a microcentrifuge tube. This process was repeated three times for each canal, resulting in a final volume of bacteria collected in PBS of 100 ml, considering both canals of each specimen^17^.

After obtaining the S1 samples, specimens of each group were submitted to the respective supplementary disinfection method investigated. Following this, the root canals were flushed with 1 ml of a 10% sodium thiosulfate solution to neutralize the effects of NaOCl. Subsequently, each canal was prepared for the collection of S2 samples and carried out in the same manner described for S1^17^.

### MTT assay

Bacterial specimens (S1 and S2) retrieved from the canals underwent a standard MTT analysis to identify the viable bacteria. Ten microliters of 3-[4,5-dimethylthiazol-2-yl]-2,5-diphenyltetrazolium bromide (MTT) reagents (Sigma-Aldrich, Steinheim, Germany) were introduced into each of the microcentrifuge tubes containing the bacterial samples. The samples were agitated and subsequently kept at 37 °C for 4 h. Afterward, 110 ml of isopropanol/HCl was included in each tube to dissolve the formazan dye. The microcentrifuge tubes were centrifuged for 5 min at 6000 rpm, and 190 ml of the supernatant was transferred to a 96-well plate. The optical density was measured at 570 nm using a SpectraStar Nano spectrophotometer (BMGLabTech, Ortenberg, Germany) (BMGLabTech, Ortenberg, Germany)^17^.

### CLSM

Confocal laser scanning microscopy (CLSM) was applied to assess the efficacy of the active supplementary disinfection methods in eradicating bacteria from the dentinal tubules of the main root canals and isthmus. For this phase, forty-four specimens (10 per group) were submitted to the same procedures described before. Four specimens were designated as controls. Two underwent autoclaving (negative control), while the others did not receive any disinfection method (positive control). After the same bacterial inoculation period, the specimens were decorated, and roots were longitudinally separated into two halves, following the procedure described by Al Shahrani et al.^23^ Specifically, a groove was created along the teeth's longitudinal axis without breaching the root canals and a chisel was employed to split each root.

**Figure 1:**
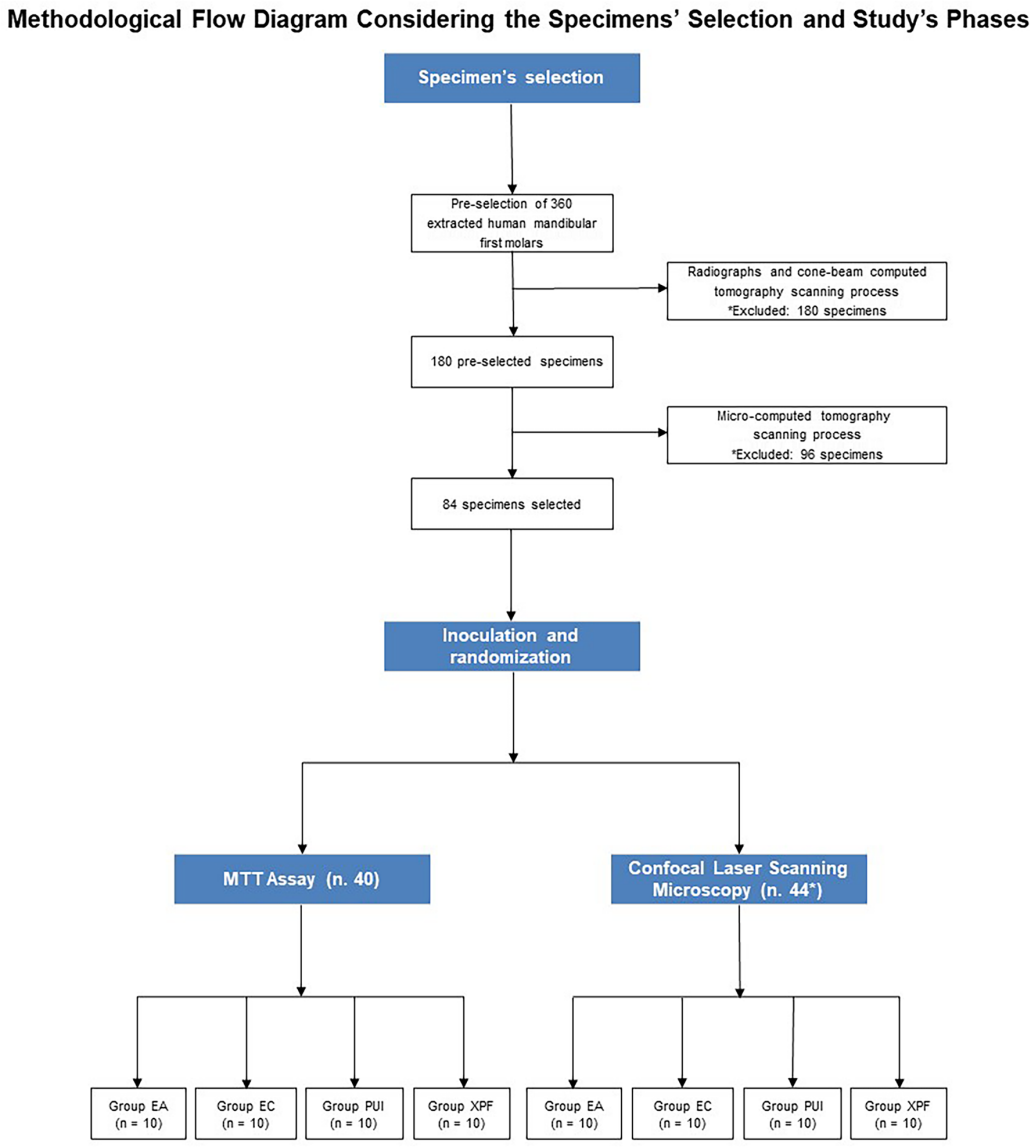
Methodological flow diagram considering the specimens’ selection and study’s phases. *Four specimens were used as control.

Subsequently, all specimens were stained using the Live/Dead Back Light bacterial viability kit (Molecular Probes, Eugene, Orange, United States) for 15 min. Then, they were submitted to the CLSM process to obtain the respective images by using a Zeiss LSM 510 confocal microscope (Carl Zeiss, Oberkochen, Germany) programmed with excitation/emission wavelengths of 480/500 nm and 490/635 nm for SYTO 9 and propidium iodide, respectively. The inspection used a 20× magnification with an additional zoom of 2×. CLSM images were captured using Zen V. 2 software (Carl Zeiss) at a resolution of 1024 × 1024 pixels. Figure 1 illustrates the methodological flow diagram considering the specimens' selection and the study's phases.

### CLSM analysis

**Figure 2:**
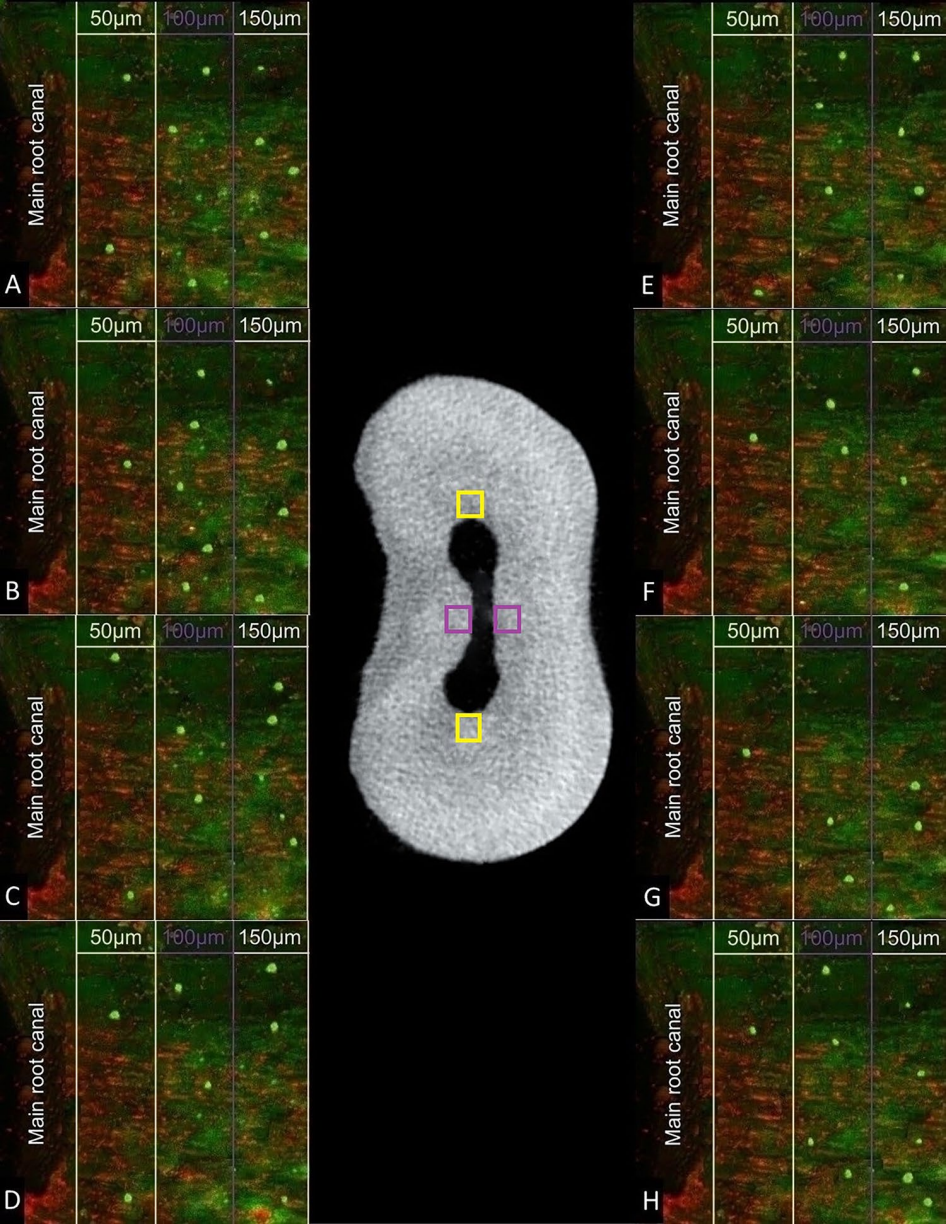
Illustrative figure showing microorganisms at different depths in dentinal tubules. Central image: cross-sectional microtomographic slice evidencing approximate locations for obtaining images of dentinal tubules from the main canals (yellow) and isthmuses (purple). CLSM images on the left and right (dentinal tubules from the main canals and isthmuses, respectively). Groups: A and E) EndoActivator. B and F) Easy Clean. C and G) Passive ultrasonic irrigation. D and H) XP-Endo Finisher.

For each specimen, 1 mm from each root canal third was scanned using a degree depth of 10 µm between each slice, resulting in 10 and 30 slices per root canal third and specimen, respectively. The ten slices from each root canal third were superposed to build up a single image from these three regions. Three distinct measurements were conducted for every superposed image, corresponding to three different depth levels (50, 100, and 150 µm into the dentinal tubules). The Zen 2 software (Zeiss) measured all the points along the dashed line within the specified depth level. At each depth level, the software quantified the intensities of red and green fluorescence (dead and live bacteria, respectively) (Fig. 2).

### Statistical analysis

All data were tabulated and subjected to statistical analysis using IBM Statistics SPSS 25.0 software (IBM, Chicago, IL, USA) with a significance level set at 0.05^24^. For the MTT assay, each group's approximated percentage of bacterial reduction was calculated using the following formula: S2 − S1/100%. For CLSM analyses, the percentage of red to red-green combined was calculated for each group through the following formula: intensity of red/intensity of red + intensity of green × 100. In both assessments, the data distribution did not show normality, according to the Kolmogorov–Smirnov normality test. Therefore, the non-parametric Kruskal–Wallis’s test was used, followed by the non-parametric pairwise Dunn multiple comparisons test. The power analysis of the test indicated that the sample size was adequate for both MTT (90%) and CLSM analyses (99%)^25^.

## Results

Considering the median of the data provided by the MTT assay, the highest percentage of bacterial reduction was obtained by XPF (94.73%), followed by PUI (82,01%) and EA (69.47%) without statistically significative differences among them (P > 0.05). A significative statistical difference was identified only when XPF (94.73%) was compared with EC (49.59%) (P = 0.017) (Table 2).

**Table 2:** Group, median, standard deviation, and standard error regarding the percentages of bacterial reduction by MTT.

Group (n. 10)	Median (−/+)	SD	SE
EA	69.47^AB^ (22.95–99.81)	26.04	8.23
EC	49.59^A^ (25.77–92.27)	22.60	7.15
PUI	82.01^AB^ (25.64–100.00)	29.43	9.31
XPF	94.73^B^ (76.04–100.00)	8.97	2.84

Different superscripted capital letters indicate statistically significant differences between the groups determined by applying Dunn's Test (P < 0.05).

(−/+): minimum and maximum values, respectively.

SD: standard deviation; SE: standard error.

Considering the percentage of the bacterial reduction provided by the CLSM analysis at 50 µm in-depth, no relevant statistical differences were identified among the groups, regardless of the place (dentinal tubules from the main canals or isthmus) (P > 0.05). In deeper depths, XPF showed better performances. In the dentinal tubules from the main root canals, at 100 and 150 µm in-depths, significant statistical differences were only observed between XPF and EC (P = 0.027) for the former and between XPF and EC (P = 0.011) and XPF and PUI (P = 0.021) for the latter. In the dentinal tubules from the isthmus, at 100 µm in-depth, statistically relevant differences did occur between XPF and EC (P = 0.038) and EC and EA (P = 0.029). At 150 µm in-depth, these differences were only significant by comparing XPF and PUI (P = 0.025) and XPF and EC (P = 0.036) (Table 3).

**Table 3:** Area, depth, group, median, standard deviation, maximum and minimum limit of the percentages of bacterial reduction by CLSM.

Area	n	Depth (µm)	EA			EC			PUI			XPF		
			Median (−/+)	SD	SE	Median (−/+)	SD	SE	Median (−/+)	SD	SE	Median (−/+)	SD	SE
DTNMC	20	50	85.50^A^ (0.00/100.00)	43.43	10.85	28.50^A^ (1.00/100.00)	33.91	8.47	38.50^A^ (3.00/99.00)	32.84	8.21	75.00^A^ (2.00/100.00)	31.13	7.78
		100	22.50^AB^ (0.00/100.00)	38.03	9.50	17.00^A^ (0.00/52.00)	17.50	4.37	24.00^AB^ (1.00/91.00)	28.95	7.23	59.50^B^ (0.00/100.00)	39.14	9.78
		150	16.50^AB^ (0.00/100.00)	39.66	9.91	13.00^A^ (0.00/63.00)	22.29	5.57	14.00^A^ (2.00/57.00)	15.66	3.91	51.50^B^ (0.00/100.00)	38.02	9.50
DTNI	20	50	53.50^A^ (4.00/100.00)	34.86	8.71	31.50^A^ (7.00/100.00)	30.48	7.62	53.00^A^ (1.00/100.00)	35.17	8.79	64.50^A^ (3.00/96.00)	35.30	8.82
		100	50.00^A^ (1.00/100.00)	38.84	9.71	11.00^B^ (0.00/97.00)	29.20	7.30	23.00^ABC^ (0.00/86.00)	25.33	6.33	42.00^AC^ (0.00/100.00)	38.40	9.60
		150	23.00^AB^ (0.00/100.00)	34.53	8.63	6.50^B^ (0.00/97.00)	31.02	7.75	8.50^B^ (0.00/74.00)	24.26	6.06	42.00^A^ (0.00/99.00)	35.54	8.88

Different superscripted capital letters indicate statistically significant differences between the groups within the two areas when comparing the three depths, determined by applying the Dunn's Test (P < 0.05).

(−/+): minimum and maximum values, respectively.

SD: standard deviation; SE: standard error.

No difference was found in the performance of the active supplementary irrigation methods compared to the results observed in the main root canals and isthmus, regardless of the depth level (P > 0.05).

## Discussion

Significant advances in metallurgical science in the last decades have impacted Endodontics by developing many instruments with different designs, purposes, manufacturing methods/surface treatments, and characteristics. However, deficiencies in the (mechanical) disinfection process have been frequently demonstrated, mainly by the relevant amounts of untouched walls in the root canals^26,27^. Hence, an increasing number of studies have been conducted to enhance the effectiveness of irrigation, primarily through different alternatives capable of promoting the agitation of the irrigating and chelating solutions due to the well-known limitations of CI^28^. This study sought to evaluate the effects of four active supplementary irrigation methods (EA, EC, PUI, and XPF) on the bacterial reduction in the main root canals by MTT assay and dentinal tubules from the main root canals and isthmus in mesial roots of extracted human mandibular first molars by CLSM. The null hypothesis was rejected because (i) XPF presented better results than EC considering the percentage of bacterial reduction provided by the MTT assay, and (ii) CLSM analyses showed that in the dentinal tubules from the main root canals, at 100 and 150 µm in-depths, significant statistical differences were observed between XPF and EC for the former and between XPF and EC, and XPF and PUI for the latter. In the dentinal tubules from the isthmus, at 100 µm in-depth, statistically relevant differences occurred between XPF, EC, and EC and EA. At 150 µm in-depth, these differences were only significant by comparing XPF, PUI, and XPF and EC.

Although endodontic infections are polymicrobial^29^, a single species culture of *E. faecalis* was selected to carry out the present research due to its following features: frequent involvement in treatment-resistant cases^30^ and in assessing materials' antimicrobial properties against endodontic infections^30^, high capacity of penetration into dentinal tubules and biofilm formation making it more resistant to disinfection processes^31^, adherence to dentin collagen^32^ and resistance to centrifugation^33^. Furthermore, *E. faecalis* can achieve a viable but unculturable state by activating a starvation response under stress conditions, which allows its regrowth^34^.

Data provided by the MTT assay showed the highest percentage of bacterial reduction was obtained by XPF (94.73%), followed by PUI (82.01%) and EA (69.47%) without statistically significant differences among them. However, XPF performed significantly better than EC (49.59%). This finding may be correlated to the design of the instruments. While EC is a straight plastic tip, XPF is a file that expands at body temperature, allowing it to provide a better debridement of the RCS.

At 50 µm in-depth, CLSM analyses showed no differences among the groups in the percentage of bacterial reduction regardless of the place (dentinal tubules from the main canals and isthmus). Nonetheless, XPR showed better performances in deeper depths. In the dentinal tubules from main canals, at 100 and 150 µm in-depth, significant statistical differences were only observed between XPF and EC for the former and between XPF and EC, and XPF and PUI for the latter. In the dentinal tubules from the isthmus, at 100 µm in-depth, statistically relevant differences occurred between XPF, EC, and EC and EA. At 150 µm in-depth, these differences were only significant by comparing XPF, PUI, and XPF and EC.

Poor results reached by EC in dentinal tubules (regardless of its location—main canals or isthmus) may be explained by the same reasons previously exposed to justify its limited performance in the main root canals. However, deficient results generally showed by PUI compared with XPF in dentinal tubules from both places analyzed (main canals and isthmus) must be discussed. While some papers showed better results of PUI over several other irrigation methods^35–37^, others found the contrary^38–40^, considering several types of analyses. The primary mechanism of action promoted by PUI to increase the cleaning and disinfection processes is the transmission of acoustic energy through the irrigating and chelating solutions, resulting in cavitation and microstreaming. This allows the solutions to move dynamically and thoroughly within the RCS. Acoustic waves generate cavitation bubbles, and the energy released upon bubble collapse is transferred to the root canal walls, initially dislodging the debris. Then, microstreaming promotes its removal from the root canal. The efficient impact of PUI is attributed to the generation of nodes along activated files, leading to the production of a strong current along the activated instrument. Multiple nodes along the instrument prevent a reduction in acoustic streaming when the file comes into contact with the canal wall. However, while microstreaming is a biophysical force strongly linked to endodontic files, the role of cavitation in vivo is controversial. The combination of acoustic streaming and cavitation can be considered a critical element in the most effective utilization of PUI^15,28,41,42^.

Even considering there is "common sense" regarding the importance of PUI to enhance the cleaning and disinfection of the RCS, recently published meta-analyses and systematic reviews have raised severe questions on the matter^43,44^. According to Moreira et al.^43^, the levels of evidence comparing PUI and CI are fragile since, in all studies researched, some bias was observed, which may interfere with the respective results and conclusions. Still, following Silva et al.^44^, there is no robust scientific evidence to effectively prove that PUI can provide better disinfection processes or increase the endodontic prognosis compared to non-activated irrigation techniques.

Considering that no other antimicrobial strategy was employed in the current research, the disinfection of the dentinal tubules could only be achieved by the penetration of the irrigating solution. In the Akcay et al.^45^ study, PUI reached a significantly larger penetration than CI, considering the sum of the penetration areas of 5.25% NaOCl at 2, 5, and 8 mm from the apex. On the other hand, Gu et al.^46^ reported no significant difference between the same techniques and areas by using 5% NaOCl. From the antimicrobial point of view, Neelakantan et al.^47^ observed no significant differences between PUI and CI in reducing *E. faecalis* biofilms from dentinal tubules at 200 and 400 µm in-depth, using 3% NaOCl followed by 17% EDTA. Azim et al.^17^ investigated the bacterial reduction from root canals and dentinal tubules provided by four irrigation methods (CI, EA, XPF, and erbium: yttrium aluminum garnet laser [PIPS]) by using the same methodological analyses employed herein. In the main root canal, MTT assays showed that bacterial reduction ranged from 89.6 to 98.2% among the groups, but XPF presented better results. In the dentinal tubules, CLSM showed that XPF promoted the more significant bacterial reduction in the three root canal thirds at 50 µm in-depth. At 150 µm in-depth, the better results were reached by PIPS. Considering PIPS was not used in the present research, in a way, our findings are in line with those obtained by Azim et al.^17^. According to Alves et al.^48^, the design and helical movement of XPF may favor the instrument to reach previously untouched areas and disrupt bacterial biofilms. However, distinct findings of the studies mentioned above may be attributed to several factors, such as different methodological designs, teeth used (anatomical complexity), microbial features (single or polymicrobial bacterial cultures; incubation/inoculation times), chemomechanical protocols, and so forth^49^.

One of the most critical advantages of performing in vitro studies is the possibility of controlling the variables to carry out more accurate investigations and thus obtain more reliable findings. Anatomical complexity plays a crucial role in endodontic research because their impacts cannot be entirely controlled but only limited. Micro‑CT is a nondestructive approach to reconstructing samples on a micrometric and real scale^50^. It is considered the gold standard research device in Endodontics for studying several matters, such as the quality of shaping and cleaning processes^51^, the performance of different obturation techniques^52^, the removal of root canal filling materials^53^, and the root canal anatomy^54^. Therefore, the main strength of the present research was the methodology developed to select the sample that has used both CBCT and micro-CT scanning processes to limit (even if partially) the impacts of the anatomical complexity on the variables studied. Even so, the standardization of dentin morphology (quantity, diameter, and depth of tubules, as well as the presence and quantity of intertubular dentin) could not be established, being, therefore, the main limitation of this scientific investigation and the most important reason to justify that its findings must be carefully transposed to the clinical practice.

The mechanical, chemical, and physical actions performed by the instrumentation and irrigation of the RCS present limitations, mainly imposed by the anatomic complexity and the microbial survivability that make it impossible to eliminate the endodontic infection^55^. The results of the present research confirm this fact since none of the irrigation protocols investigated provided total microbial elimination from both sites studied (main root canal and dentinal tubules). Therefore, new and more effective methods to disinfect the RCS should be sought, as the presence of microorganisms at the filling time does not necessarily cause failure; however, its absence certainly favors the success of endodontic treatment^55^.

## Conclusions

Regarding the limitations of the present research, no irrigation method could render the RCS thoroughly disinfected. Nonetheless, XPF presented better results than the other systems in both analyses (MTT assay and CLSM).

## Data Availability

The datasets and the complete statistical analysis of the present research are available and can be requested from the corresponding author.
